# Genetic predisposition to high circulating levels of interleukin 6 and risk for Alzheimer's disease. Discovery and replication

**DOI:** 10.1016/j.tjpad.2024.100018

**Published:** 2025-01-01

**Authors:** Sokratis Charisis, Niki Mourtzi, Matthew R. Scott, Eva Ntanasi, Eirini Mamalaki, Alexandros Hatzimanolis, Alfredo Ramirez, Jean-Charles Lambert, Mary Yannakoulia, Mary Kosmidis, Efthimios Dardiotis, Georgios Hadjigeorgiou, Paraskevi Sakka, Claudia L Satizabal, Alexa Beiser, Qiong Yang, Marios Κ. Georgakis, Sudha Seshadri, Nikolaos Scarmeas

**Affiliations:** a Glenn Biggs Institute for Alzheimer's and Neurodegenerative Diseases, UT Health San Antonio, San Antonio, TX, USA; b 1st Department of Neurology, Aiginition Hospital, National and Kapodistrian University of Athens Medical School, Athens, Greece; c Department of Biostatistics, Boston University School of Public Health; d Department of Nutrition and Dietetics, Harokopio University, Athens, Greece; e Department of Psychiatry, Aiginition Hospital, National and Kapodistrian University of Athens Medical School, Athens, Greece; f Division of Neurogenetics and Molecular Psychiatry, Department of Psychiatry and Psychotherapy, University of Cologne, Medical Faculty, Cologne, Germany; g Department of Neurodegenerative Diseases and Geriatric Psychiatry, University Hospital Bonn, Bonn, Germany; h German Center for Neurodegenerative Diseases (DZNE Bonn), Bonn, Germany; i Excellence Cluster on Cellular Stress Responses in Aging-Associated Diseases (CECAD), University of Cologne, Cologne, Germany; j Univ. Lille, Inserm, CHU Lille, Institut Pasteur de Lille, U1167-RID-AGE facteurs de risque et déterminants moléculaires des maladies liés au vieillissement, Lille, France; k Lab of Cognitive Neuroscience, School of Psychology, Aristotle University of Thessaloniki, Thessaloniki, Greece; l Department of Neurology, University Hospital of Larisa, Faculty of Medicine, School of Health Sciences, University of Thessaly, Larisa, Greece; m Department of Neurology, Medical School, University of Cyprus, Nicosia, Cyprus; n Athens Association of Alzheimer's Disease and Related Disorders, Marousi, Greece; o Center for Genomic Medicine, Massachusetts General Hospital, Boston, MA, USA; p Program in Medical and Population Genetics, Broad Institute of Harvard and the Massachusetts Institute of Technology, Boston, MA, USA; q Institute for Stroke and Dementia Research (ISD), University Hospital, Ludwig-Maximilians-University (LMU) Munich, Munich, Germany; r Department of Neurology, The Gertrude H. Sergievsky Center, Taub Institute for Research in Alzheimer's Disease and the Aging Brain, Columbia University, New York, NY, USA

**Keywords:** Interleukin 6, Polygenic risk score, Alzheimer's disease, Mild cognitive impairment, Dementia

## Abstract

**Importance:**

Aging is accompanied by immune dysregulation, which has been implicated in Alzheimer's disease (AD) pathogenesis. Individuals who are genetically predisposed to elevated levels of proinflammatory mediators might be at increased risk for AD.

**Objective:**

To investigate whether genetic propensity for higher circulating levels of interleukin 6 (IL-6) is associated with AD risk.

**Design:**

We analyzed data from the Hellenic Longitudinal Investigation of Aging and Diet (HELIAD). Mean follow-up was 2.9 (SD, 0.8) years. Baseline assessment was from 11/2009 to 11/2016, and cognitive follow-up from 01/2013 to 07/2019. Associations of interest were also examined in the UK Biobank (UKB) for replication purposes (mean follow-up was 12.9 (SD, 2.4) years; baseline assessment was from 12/2006 to 10/2010).

**Setting:**

Population-based study.

**Participants:**

The HELIAD sample included 622 participants ≥65 years of age without baseline dementia or amnestic mild cognitive impairment (aMCI-the prodromal stage of AD). The UKB sample included 142,637 participants ≥60 years of age without prevalent dementia.

**Exposures:**

Genetic predisposition to elevated circulating levels of IL-6 was estimated using a polygenic risk score (PRS), calculated based on the summary statistics of a current GWAS meta-analysis.

**Main Outcomes and Measures:**

AD and MCI diagnoses were based on standard clinical criteria [HELIAD], or hospital records and death registry data [UKB]. Associations with AD or aMCI incidence [HELIAD] and AD incidence [UKB] were examined with Cox regression models.

**Results:**

In HELIAD, mean age was 73.4 (SD, 5.0) years; 363 (58%) women. An increase in IL-6 PRS by 1 standard deviation unit (SDU) was associated with up to a 43% increase in the risk for incident AD/aMCI (HR_GWAS significance threshold of 0.01,_ 1.43 [95%CI, 1.14 – 1.80]). In UKBB, mean age was 64.2 (SD, 2.8) years; 73,707 (52%) women. A 1 SDU increase in IL-6 PRS was associated with up to an 8% increase in the risk for incident AD (HR_GWAS significance threshold of 0.2_, 1.08 [95%CI, 1.04 – 1.12]).

**Conclusions and Relevance:**

Genetic predisposition to higher circulating levels of IL-6 was associated with an increased risk for AD, supporting the role of IL-6-related pathways in AD pathogenesis, and suggesting that genetic predisposition to proinflammatory states might trigger or accelerate AD-related neuropathology.

## 1 Introduction

Aging is accompanied by physiological changes in the immune system, collectively known as immunosenescence [1]. One of the hallmarks of immunosenescence is the institution of a chronic low-grade systemic inflammatory state, characterized by elevated circulating levels of pro-inflammatory mediators, including interleukin (IL)−1, IL-6, and tumor necrosis factor (TNF), a process often referred to as inflammaging [2]. Inflammaging has been linked to an increased risk for many chronic diseases, including cardiovascular disease, cancer, and, from a brain standpoint, cognitive decline and dementia [[2], [3], [4]].

Genetic predisposition to overactive immune responses might, at least partially, explain why in some older individuals inflammaging remains a subclinical aging-associated phenotype, while in others it might contribute to the development of neurodegenerative conditions, such as Alzheimer's disease (AD) [4]. This hypothesis is supported by prior findings relating certain genetic polymorphisms in genes encoding pro-inflammatory cytokines, such as IL-1 and IL-6, with the risk for AD [[5], [6], [7], [8]]. IL-6 appears to be involved in AD pathophysiology, potentially intersecting inflammatory and neurodegenerative pathways. Increased IL-6 mRNA expression has been found in brain areas where amyloid deposition and astroglia activation are prominent in individuals with AD [9,10], and increased IL-6 levels in the brain have been implicated in early stages of plaque formation [11].

Nevertheless, the study of individual genetic variants provides limited information in the setting of polygenic traits, such as those related to immune system functions and inflammation [12], as effect sizes for the majority of individual variants are typically negligible, and the fraction of true causal variants among them is small [13]. Methods aggregating the effects of single variants across the entire genome might more accurately capture the heritability of complex traits. Among them, polygenic risk scores (PRSs) have emerged as particularly useful tools, due to their ability to predict disease status in a variety of research settings [14]. To our knowledge, studies leveraging these tools to comprehensively assess an individual's genetic propensity to inflammation and identify potential associations with the risk for AD are lacking.

In the present work, using the summary statistics of a genome-wide meta-analysis [15], we calculated a PRS to estimate genetic predisposition to increased circulating levels of IL-6, and studied its association with incident AD or its prodromal stage, amnestic mild cognitive impairment (aMCI) [16], in two independent prospective cohorts of community-dwelling individuals without dementia.

## 2 Methods

### 2.1 Participants

**Fig. 1:**
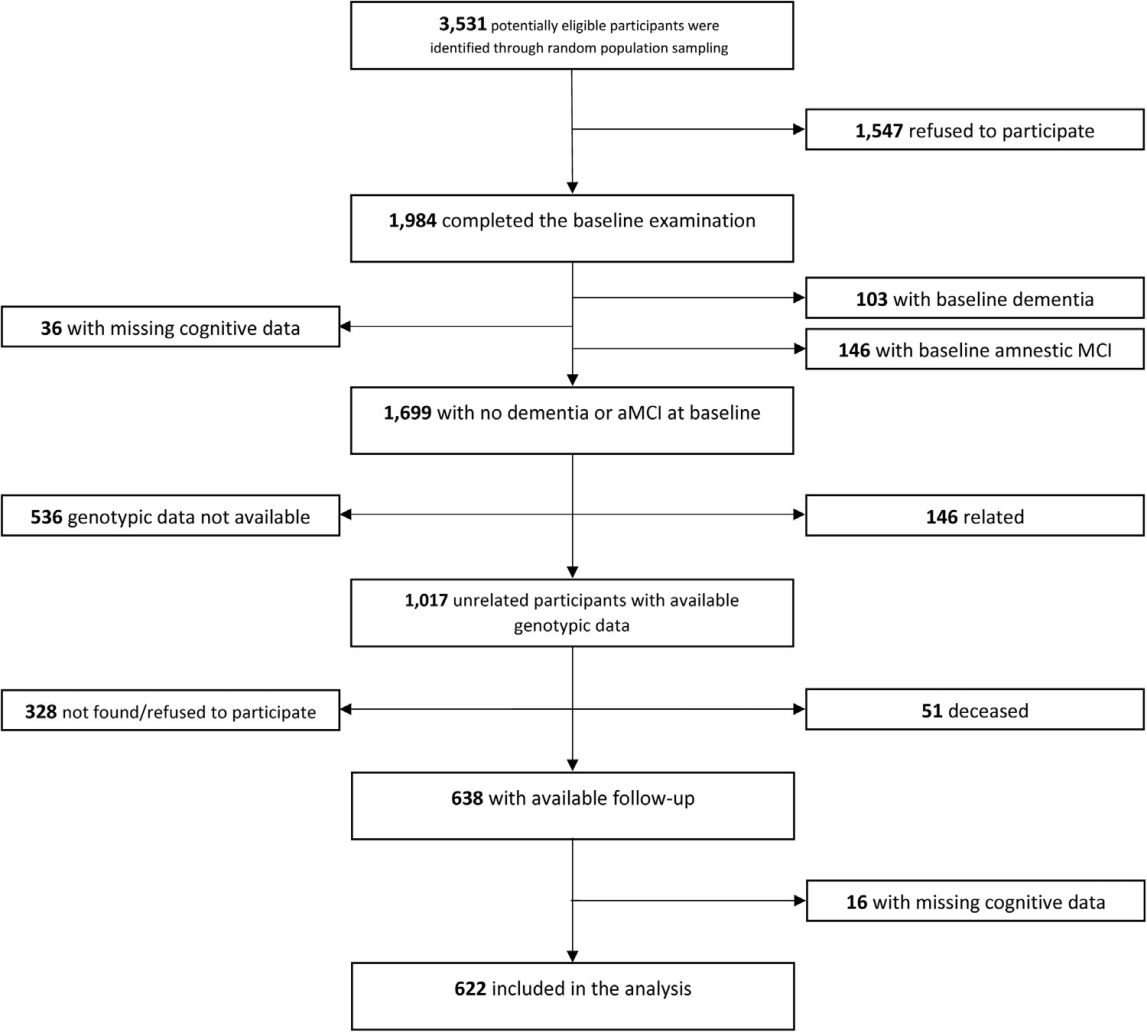
Flowchart of study sample.

The Hellenic Longitudinal Investigation of Aging and Diet (HELIAD) is an ongoing population-based study of individuals ≥65 years of age, recruited from the general population of two Greek districts through random sampling [[17], [18], [19]]. Baseline demographic, cognitive, and genotypic data were collected from 11/2009 to 11/2016. Cognitive follow-up data were collected from 01/2013 to 07/2019. The present prospective analysis included 622 participants based on the following inclusion criteria: (i) no baseline dementia or aMCI, (ii) available cognitive follow-up, (iii) available demographic and genotypic data (Fig. 1).

### 2.2 Diagnostic criteria

Diagnoses were reached through diagnostic consensus meetings of all the main investigators, both neurologists and neuropsychologists, as previously described [20,21]. Dementia was diagnosed based on the DSM-IV-TR criteria [22], and the designation of probable or possible AD was made according to the National Institute of Neurological and Communicative Disorders and Stroke/Alzheimer Disease and Related Disorders Association (NINCDS/ADRDA) criteria [23]. The diagnosis of MCI was based on the Petersen criteria [24]; MCI was subclassified as amnestic, in cases of isolated memory impairment or multi-domain cognitive impairment involving memory, and as non-amnestic, in cases of isolated or combined impairment of other cognitive domains (i.e., language, attention, executive function, visuo-perceptual) with memory sparing.

### 2.3 Genotyping, imputation, and PRS calculation

The methodology of genome-wide genotyping, quality control, and imputation has been described elsewhere [20,25], and is also included in the Supplement (eMethods 1). Genetic predisposition to elevated circulating levels of IL-6 was modeled through a PRS, calculated using the summary statistics from a recent genome-wide meta-analysis [15], including a total of 21,758 individuals from 13 cohorts of European ancestry.

First, imputed dosages for a total of 5,611,082 single nucleotide polymorphisms (SNPs) with minor allele frequency (MAF) >0.05, call rate >95%, and imputation quality score >0.4 were converted to best-guess genotypes (with probability >0.8). Then, the PRSice software (http://prsice.info/) was used for PRS calculation by applying the clumping and thresholding (C + T) method [26,27]. A risk score was calculated for each SNP by multiplying the risk allele number (i.e., 0, 1, 2) with the corresponding effect size (i.e., beta coefficient) reported in the GWAS summary data. A set of PRSs were then computed for each participant by summing the individual SNP-risk scores for SNPs achieving genome-wide significance at 10 a priori-defined GWAS p-value thresholds (i.e., 5 × 10^−8^, 0.0001, 0.001, 0.01, 0.05, 0.1, 0.2, 0.3, 0.4, 0.5). The number of SNPs included in PRS calculation at each GWAS significance threshold is reported in eTable 1. Higher PRS scores reflect greater genetic predisposition for elevated plasma levels of IL-6. To ensure that only independent markers were included, SNP clumping based on linkage disequilibrium (LD) was performed, using the default PRSice settings for clumping (r_c_^2^ of 0.1 and w_c_ of 250 kb). SNPs located within the *APOE* region, defined as 1 Mb up and down-stream of the *APOE* gene (chromosome 19: 44.4–46.5 Mb), were excluded from PRS calculation, and the *APOE* genotype (coded as presence or absence of ε4 allele) was included as predictor in the analyses, as previously proposed [28].

### 2.4 Vascular burden score calculation

To assess the vascular burden of study participants, we computed a vascular burden score (VBS), as previously described [[29], [30], [31]]. VBS for each participant was the sum of the following cardiovascular risk factors and diseases: (i) hypertension (according to past history, medical records, or antihypertensive medication use), (ii) diabetes mellitus (according to past history, medical records, or glucose-lowering medication use), (iii) hyperlipidemia (according to past history, medical records, or lipid-lowering medication use), (iv) heart disease (according to past history of ischemic heart disease, myocardial infarction, coronary angioplasty, coronary artery bypass surgery, congestive heart failure, atrial fibrillation or other arrhythmias, or pacemaker implantation), and (v) cerebrovascular disease (according to past history, or prior symptoms suggestive of stroke or transient ischemic attack). Each score component was assigned a score of 1; the VBS score ranged from 0 to 5.

### 2.5 Statistical analysis

Normality of data was graphically explored using Q-Q plots and Kernel density plots. Participant characteristics were compared using ANOVA and Kruskal-Wallis tests for normally and non-normally distributed continuous variables, respectively, and Pearson's chi-square for categorical variables. Variables expressing PRS scores were converted to z-scores before entered in survival analysis models.

#### 2.5.1 Primary analyses

Associations of IL-6 PRS with the composite outcome of incident AD or aMCI were explored using Cox proportional hazards models. Time-to-event was time from baseline evaluation to visit of AD or aMCI diagnosis; participants who did not develop AD or aMCI were right-censored at the time of their last evaluation. The calculated PRS scores at different GWAS significance thresholds were the main predictors. Two sets of models were constructed for each threshold: (i) Model 1 was adjusted for baseline age, sex, study center, years of education, *APOE* genotype, presence of non-amnestic MCI, and the first two principal components for population structure (to control for potential cryptic relatedness and unexpected genotyping batch errors); (ii) Model 2 was further adjusted for VBS. The proportional hazards assumption was tested using Schoenfeld residuals [32]; for variables violating the proportional hazards assumption we included time-varying coefficients by specifying interaction terms with an identity function for analysis time [33]. For illustration purposes, we also categorized participants into low (> 1 SD below the mean), intermediate (within 1 SD of the mean), and high (> 1 SD above the mean) IL-6 PRS groups, and generated adjusted survival curves for each group (eMethods 3).

#### 2.5.2 Replication

To examine the replicability and external validity of primary analysis findings, we studied the associations of IL-6 PRS with AD incidence in the UK Biobank (UKB), a large prospective population-based cohort of participants aged 40–69 years, recruited between 2006 and 2010 in the UK [34]. The replication sample consisted of 142,637 unrelated participants ≥60 years of age without prevalent dementia. Participants attended the baseline evaluation between 12/2006 and 10/2010. Dementia cases were identified using hospital inpatient admission records and death registry data, available from the Hospital Episode Statistics for England, the Scottish Morbidity Record for Scotland, and the Patient Episode Database for Wales. Further details are included in the Supplement (eMethods 2). Analyses were similarly structured to those described in Section 2.5.1. Specifically, Cox models adjusted for baseline age, sex, education, *APOE* genotype, and the first two principal components for population structure were computed (Model 1). The outcome was restricted to incident AD since information on MCI subclassification was not available. In sensitivity analyses, we computed models further adjusted for Townsend deprivation index to account for material deprivation (Model 2). Further, we conducted competing-risk analysis with death as a competing event using the Fine-Gray subdistribution hazard model [35].

The false discovery rate (FDR) was controlled at <5% using the Benjamini-Hochberg procedure [36], to account for testing of multiple GWAS significance thresholds. All analyses were performed using R, version 4.2.3 (R Foundation for Statistical Computing, 2023). A 2-sided p-value of <0.05 was considered statistically significant.

## 3 Results

### 3.1 Participant characteristics in HELIAD

Missing data analyses are reported in the Supplement (eResults 1, eTable 2, eTable 3). Out of 622 participants included in the analysis, 73 developed AD or aMCI over a mean follow-up of 2.9 (SD, 0.8) years. Mean age was 73.4 (SD, 5.0) years, and 363 (58%) participants were women (Table 1). Participants who developed AD or aMCI were older, less educated, had a higher prevalence of non-amnestic MCI at baseline, and had higher IL-6 PRS scores at 7 out of 10 GWAS significance thresholds, compared to those who remained AD/aMCI-free. There were no differences in sex, study center, VBS, or *APOE* ε4 carrier status between participants with and those without incident AD or aMCI.

**Table 1:** HELIAD participant baseline characteristics by AD or amnestic MCI incidence.

	Total (622)	No AD or amnestic MCI at follow-up (549)	AD or amnestic MCI at follow-up (73)	P value
Age, years, mean (SD)	73.4 (5.0)	73.2 (4.9)	75.1 (5.4)	**0.002**^a^
Sex, n (%) Women Men	363 (58) 259 (42)	323 (59) 226 (41)	40 (55) 33 (45)	0.51
Study center, n (%) Larissa Marousi	556 (89) 66 (11)	492 (90) 57 (10)	64 (88) 9 (12)	0.61
Education, years, median (IQR)	6 (5, 11)	6 (6, 11)	6 (3, 7)	**<0.001**^a^
Vascular burden score, median (IQR)	2 (1, 2)	2 (1, 2)	2 (1, 2)	0.90
*APOE* ε4 allele carrier, n (%) No Yes	524 (84) 98 (16)	463 (84) 86 (16)	61 (84) 12 (16)	0.86
Non-amnestic MCI, n (%) No Yes	593 (95) 29 (5)	528 (96) 21 (4)	65 (89) 8 (11)	**0.007**^a^
PRS for IL-6, mean (SD) GWAS threshold 5 × 10^−8^ GWAS threshold 0.0001 GWAS threshold 0.001 GWAS threshold 0.01 GWAS threshold 0.05 GWAS threshold 0.1 GWAS threshold 0.2 GWAS threshold 0.3 GWAS threshold 0.4 GWAS threshold 0.5	−437.91 (204.13) −1.47 (20.08) 9.74 (6.52) −3.25 (2.00) 0.33 (0.91) −0.62 (0.65) 0.55 (0.47) −0.09 (0.38) 0.10 (0.34) 0.09 (0.31)	−441.02 (204.03) −1.85 (20.20) 9.75 (6.52) −3.31 (1.99) 0.30 (0.92) −0.65 (0.65) 0.54 (0.47) −0.10 (0.38) 0.09 (0.34) 0.08 (0.31)	−414.53 (204.77) 1.40 (19.07) 9.65 (6.54) −2.74 (2.04) 0.56 (0.84) −0.45 (0.63) 0.67 (0.46) 0.01 (0.37) 0.19 (0.34) 0.16 (0.30)	0.30 0.19 0.91 **0.02**^a^ **0.02**^a^ **0.02**^a^ **0.02**^a^ **0.02**^a^ **0.02**^a^ **0.03**^a^

Abbreviations: HELIAD, Hellenic Longitudinal Investigation of Aging and Diet; AD, Alzheimer's disease; MCI, mild cognitive impairment; SD, standard deviation; IQR, interquartile range; PRS, polygenic risk score; IL-6, interleukin 6; GWAS, genome-wide association study.

a Significant at *p* ≤.05.

### 3.2 Associations with AD or aMCI incidence in HELIAD

**Fig. 2:**
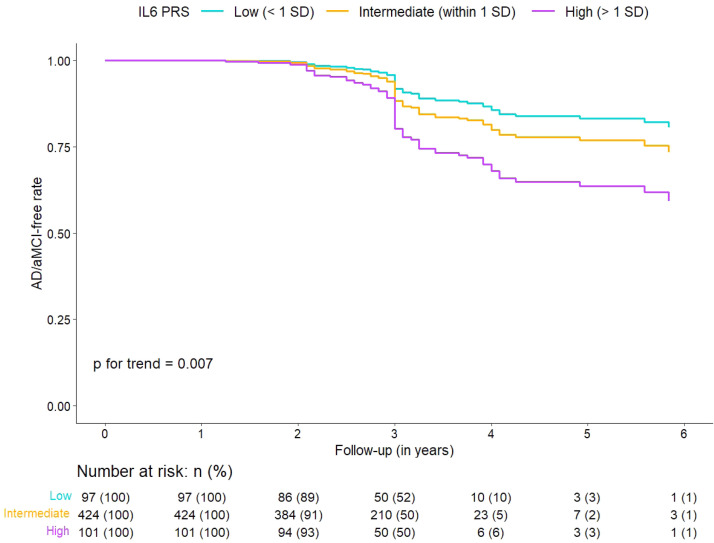
Expected survival curves calculated separately for participants in different (low, intermediate, and high) IL-6 PRS categories, based on adjusted (for age, sex, study center, years of education, *APOE* ε4 carrier status, presence of non-amnestic MCI, and the first two principal components for population structure) Cox models. Abbreviations: AD, Alzheimer's disease; aMCI, amnestic mild cognitive impairment; IL-6, interleukin 6; PRS, polygenic risk score; SD, standard deviation.

Cox regression models revealed associations of IL-6 PRS with AD or aMCI incidence at 8 out of 10 GWAS significance thresholds in the HELIAD sample (Table 2, Model 1). An increase in IL-6 PRS by 1 standard deviation unit (SDU) was associated with an increase in the risk for AD or aMCI ranging from 30% to 43%. For example, at a GWAS significance threshold of 0.01, an increase in IL-6 PRS by 1 SDU was associated with a 43% (95% confidence interval [CI], 14% to 80%) increase in the risk for incident AD or aMCI (Table 2, Model 1). These associations remained significant after FDR control. After further adjustment for VBS, the results remained largely unchanged (Table 2, Model 2). Adjusted survival curves for different (i.e., low, intermediate, and high) IL-6 PRS groups based on a GWAS significance threshold of 0.01 are presented in Fig. 2.

**Table 2:** Associations between PRS for IL-6 and AD or amnestic MCI incidence in the HELIAD cohort.

		Model 1 a				Model 2 b			
PRS for IL-6		At risk, n	HR (95% CI)	Raw p value	Adjusted p value	At risk, n	HR (95% CI)	Raw p value	Adjusted p value
Genome-wide significance threshold	5 × 10^−8^	622	**1.30 (1.03 - 1.66)**	**0.03**	**0.04**^c^	615	**1.31 (1.03 - 1.68)**	**0.03**	**0.04**^c^
	0.0001		1.24 (0.98 - 1.56)	0.07	0.08		1.25 (0.99 - 1.59)	0.07	0.07
	0.001		1.11 (0.88 - 1.41)	0.38	0.38		1.11 (0.88 - 1.42)	0.38	0.38
	0.01		**1.43 (1.14 - 1.80)**	**0.002**	**0.01**^c^		**1.43 (1.13 - 1.81)**	**0.003**	**0.02**^c^
	0.05	Events, n	**1.43 (1.13 - 1.82)**	**0.003**	**0.01**^c^	Events, n	**1.42 (1.11 - 1.80)**	**0.005**	**0.02**^c^
	0.1		**1.35 (1.07 - 1.70)**	**0.01**	**0.02**^c^		**1.33 (1.05 - 1.68)**	**0.02**	**0.03**^c^
	0.2	73	**1.34 (1.06 - 1.70)**	**0.01**	**0.02**^c^	70	**1.33 (1.04 - 1.69)**	**0.02**	**0.03**^c^
	0.3		**1.43 (1.12 - 1.82)**	**0.004**	**0.01**^c^		**1.41 (1.10 - 1.81)**	**0.008**	**0.03**^c^
	0.4		**1.37 (1.08 - 1.75)**	**0.009**	**0.02**^c^		**1.34 (1.05 - 1.72)**	**0.02**	**0.03**^c^
	0.5		**1.36 (1.07 - 1.72)**	**0.01**	**0.02**^c^		**1.33 (1.04 - 1.71)**	**0.02**	**0.03**^c^

Results from Cox proportional hazards models with AD or amnestic MCI incidence as the outcome, and IL-6 PRS as the main predictor.

a Model 1 was adjusted for baseline age, sex, study center, years of education, *APOE* ε4 carrier status, presence of non-amnestic MCI, and the first two principal components for population structure.

b Model 2 was further adjusted for vascular burden score.

c Significant after false-discovery rate control at <5% using the Benjamini-Hochberg procedure. Abbreviations: HELIAD, Hellenic Longitudinal Investigation of Aging and Diet; PRS, polygenic risk score; IL-6, interleukin 6; AD, Alzheimer's disease; MCI, mild cognitive impairment; HR, hazard ratio; CI, confidence interval.

### 3.3 Associations with AD or aMCI incidence in UKB

In the UKB sample, a total of 2,737 incident AD cases were identified over a mean follow-up of 12.9 (SD, 2.4) years. Mean age was 64.2 (SD, 2.8) years, and 52% of participants were women (eTable 4). Participants who developed AD were older, less educated, were more likely to be *APOE* ε4 allele carriers, and had higher IL-6 PRS scores at 7 out of 10 GWAS significance thresholds, compared to those who remained AD-free. There were no significant differences in sex between participants who developed incident AD and those who did not.

Cox regression models revealed associations of IL-6 PRS with AD incidence at 6 out of 10 GWAS significance thresholds after FDR control (Table 3, Model 1). A 1 SDU increase in IL-6 PRS was associated with an increase in the risk for AD ranging from 6% to 8%. For example, at a GWAS significance threshold of 0.2, an increase in IL-6 PRS by 1 SDU was associated with an 8% (95% CI, 4% to 12%) increase in the risk for incident AD. In sensitivity analyses (i.e., models further adjusted for material deprivation and competing-risk analysis with death as a competing event), the results remained virtually unchanged (Table 3, Model 2; eTable 5).

**Table 3:** Associations between PRS for IL-6 and AD incidence in the UK Biobank.

		Model 1 a				Model 2 b			
PRS for IL-6		At risk, n	HR (95% CI)	Raw p value	Adjusted p value	At risk, n	HR (95% CI)	Raw p value	Adjusted p value
Genome-wide significance threshold	5 × 10^−8^	142,637	0.99 (0.96 - 1.03)	0.678	0.753	142,521	0.99 (0.96 - 1.03)	0.649	0.722
	0.0001		0.99 (0.95 - 1.03)	0.626	0.753		0.99 (0.95 - 1.03)	0.608	0.722
	0.001		1.00 (0.97 - 1.04)	0.899	0.899		1.00 (0.97 - 1.04)	0.9	0.9
	0.01		1.04 (1.00 - 1.08)	0.038	0.055		1.04 (1.00 - 1.08)	0.042	0.06
	0.05	Events, n	**1.06 (1.02 - 1.10)**	**0.002**	**0.003**^c^	Events, n	**1.06 (1.02 - 1.10)**	**0.002**	**0.004**^c^
	0.1		**1.06 (1.02 - 1.10)**	**0.001**	**0.003**^c^		**1.06 (1.02 - 1.10)**	**0.001**	**0.004**^c^
	0.2	2,737	**1.08 (1.04 - 1.12)**	**<0.001**	**0.001**^c^	2,736	**1.08 (1.04 - 1.12)**	**<0.001**	**0.001**^c^
	0.3		**1.07 (1.03 - 1.11)**	**<0.001**	**0.002**^c^		**1.07 (1.03 - 1.11)**	**<0.001**	**0.002**^c^
	0.4		**1.06 (1.02 - 1.10)**	**0.001**	**0.003**^c^		**1.06 (1.02 - 1.10)**	**0.001**	**0.004**^c^
	0.5		**1.06 (1.02 - 1.10)**	**0.002**	**0.003**^c^		**1.06 (1.02 - 1.10)**	**0.002**	**0.004**^c^

Results from Cox proportional hazards models with AD incidence as the outcome, and IL-6 PRS as the main predictor.

a Model 1 was adjusted for baseline age, sex, education, *APOE* ε4 carrier status, and the first two principal components for population structure.

b Model 2 was further adjusted for Townsend deprivation index.

c Significant after false-discovery rate control at <5% using the Benjamini-Hochberg procedure. Abbreviations: PRS, polygenic risk score; IL-6, interleukin 6; AD, Alzheimer's disease; HR, hazard ratio; CI, confidence interval.

## 4 Discussion

In this study, genetic predisposition to higher circulating levels of IL-6 was associated with an increased risk for AD or aMCI in a sample of community-dwelling older adults. These findings were replicated in more than 142,600 individuals from an independent population-based cohort, enhancing their generalizability and external validity. The present results further support the role of inflammatory pathways in AD pathogenesis and suggest that genetic susceptibility to proinflammatory states might trigger or accelerate AD-related neuropathology.

### 4.1 Associations with AD or aMCI incidence

A case-control study including 332 individuals with probable AD and 393 controls from Italy, investigated two different polymorphic regions of the IL-6 gene [7]. The −174 C allele in the promoter region of the IL-6 gene was overrepresented in AD participants compared to controls, and was associated with an increased risk for the disease. Moreover, the −174 CC genotype was associated with a higher risk for AD in women. Additionally, the D allele of a variable number of tandem repeat (VNTR) was in strong LD with the −174 C allele. The VNTR DD genotype was more frequent in AD participants compared to controls, and was also associated with an increased risk for the disease. On the other hand, the frequency of the VNTR C allele was decreased in participants with AD, and was associated with a decreased risk for the disease. Importantly, both the −174 CC and VNTR DD genotypes were associated with increased IL-6 levels in the blood and brain of individuals with AD.

In two case-control studies conducted in Chinese populations, one including 318 individuals with AD and 324 controls [37], and the other 341 individuals with AD and 421 controls [38], the −572 G allele as well as the −572 GG genotype in the promoter region of the IL-6 gene were associated with a decreased risk for AD; in the second study this association was only present in *APOE* ε4 allele carriers [38]. A later study including 266 individuals with AD and 444 controls from Taiwan, replicated these findings but also identified an association of another SNP located in the intron region of IL-6 gene with AD risk [5]; the authors postulated that due to its intronic location, this SNP may affect disease risk through alternative splicing.

The abovementioned findings suggest a link between IL-6 genetic polymorphisms and AD risk, which might, in part, be mediated by elevated levels of circulating IL-6. Nonetheless, findings pertaining to individual variants are subject to limitations, as they usually capture, at best, only a small proportion of the genetic variation associated with complex traits [39], and the proportion of true causal variants among them is typically rather small [13].

Instead of focusing on single variant effects, a PRS aggregates the effects of independent risk variants across the entire genome to comprehensively assess the heritability of complex traits. Previous studies have shown that PRSs achieve substantially greater predictive power than a small number of SNPs [13], and they can accurately predict disease status in a variety of research settings [14]. Herein, we leveraged a PRS approach to estimate genetic susceptibility to elevated circulating levels of IL-6, and identify associations with the risk for developing AD, or its prodromal form, aMCI [16].

Interestingly, effect sizes for the associations between IL-6 PRS and AD incidence differed between the two cohorts, with relatively larger estimates observed in the HELIAD cohort (a 1 SDU increase in IL-6 PRS was associated with a 30–43% increase in the risk for AD or aMCI), compared to the UKB (a 1 SDU increase in IL-6 PRS was associated with a 6–8% increase in the risk for AD). These differences could be related to a potentially higher accuracy of the diagnostic algorithm used in the HELIAD cohort, relying on expert consensus diagnoses based on standard clinical criteria, compared to the hospital record/death registry-derived diagnoses used in the UKB [40]. The comprehensive diagnostic algorithm of the HELIAD cohort also allowed us to identify and include incident aMCI cases as events of interest, perhaps increasing outcome sensitivity, whereas such information was not available in the UKB sample. Finally, differences in the two study populations - such as the younger baseline age of UKB participants (mean age: 64.2 years) compared to HELIAD participants (mean age: 73.4 years) - and the overall better health and socioeconomic status of UKB participants relative to the general population [41], might have also contributed to the variation in results.

### 4.2 The role of vascular burden

Considering prior evidence pointing towards an association of circulating IL-6 levels with cerebrovascular events and vascular cognitive impairment [42,43], as well as towards additive or synergistic effects of vascular pathology on AD-related cognitive decline [44], we hypothesized that potential relationships of genetic predisposition for high circulating levels of IL-6 with AD pathology might be confounded, or mediated, by vascular pathways. After taking into account the vascular disease burden in our analysis, the results remained virtually unchanged, suggesting that IL-6 PRS was related to AD/aMCI incidence independent of vascular pathology.

### 4.3 Biological plausibility/putative molecular pathways

As part of the aging process, the immune system undergoes a process of senescence, characterized by physiological alterations that lead to the development of certain aging-associated phenotypes, such as decreased immune efficacy and inadequate responses against novel antigens and vaccines [1]. Additionally, the immune system begins to adversely affect human health, possibly contributing to the development and clinical course of age-related conditions, such as cardiovascular, metabolic, and neurodegenerative diseases [45]. A hallmark feature of immunosenescence is the increase in cellular production of proinflammatory cytokines, such as IL-1β, IL-6, and TNF, leading to the institution of a chronic low-grade systemic inflammatory state (inflammaging) [3]. These inflammatory mediators might access the brain, and contribute to reduced brain-derived neurotrophic factor levels, glutamatergic activation (excitotoxicity), oxidative stress, and induction of apoptosis [46].

Despite this general biological framework, the clinical picture of inflammaging can vary widely, ranging from a subclinical aging-related phenotype, to an important contributor to cardiovascular diseases and dementia [2,4]. Genetic variability might constitute one of the key factors responsible for this phenotypic heterogeneity, as individuals genetically predisposed to proinflammatory states and overactive immune responses might be inherently more prone to the detrimental effects of inflammaging [2]. Among associations between genetic variants and levels of proinflammatory mediators in the blood that might be particularly relevant with respect to AD, are those related to the IL-6 gene. Prior findings have implicated IL-6 in mainstream AD-related neurodegenerative pathways such as amyloid deposition and plaque formation [[9], [10], [11]], tau hyperphosphorylation [47], and synaptic damage [48] (eFigure 1). In addition, higher circulating levels of IL-6 have been associated with MRI markers of global brain and hippocampal atrophy [49], suggesting a link between blood IL-6 levels and AD-like brain atrophy patterns [50].

The present findings further support the involvement of IL-6-related pathways in AD pathogenesis, by suggesting that genetic predisposition to high circulating levels of IL-6 might trigger or potentiate AD-related neuropathological processes, perhaps through amplifying aging-related proinflammatory states and worsening the underlying immune dysregulation.

### 4.4 Limitations

First, circulating IL-6 levels were not available. However, the out-of-sample performance of IL-6 PRS has been previously assessed in the European Malmö Diet and Cancer study [15]. Second, we had to consider all predefined genome-wide significance thresholds in our analysis and apply corrections for multiple testing, with potential decreases in power, which could have been avoided if IL-6 levels were available to identify the PRS threshold explaining the maximum amount of trait variance in each cohort. Third, a limitation of current PRS methods is that they rely on individuals’ genetic ancestry being similar to the GWAS study from which reference effect sizes for PRS calculation are obtained [14]. This might hamper the assessment of the replicability and generalizability of the present findings to populations and ethnic groups in which such large-scale GWAS studies are not available. Fourth, biomarkers of amyloid-β and tau pathology were not available to confirm the presence of core AD neuropathology in participants who developed AD or aMCI over the study follow-up.

### 4.5 Conclusion

In summary, genetic predisposition to higher circulating levels of IL-6, assessed by leveraging GWAS results and aggregating common variant effects across the entire genome through a PRS approach, was positively associated with AD incidence in two independent population-based cohorts. Along with supporting the role of IL-6-related pathways in AD pathogenesis, these findings also highlight the importance of anti-inflammatory lifestyle, or other, interventions, to counterbalance the cognitive risk conferred by aging-associated proinflammatory phenotypes, especially in genetically predisposed individuals. Further studies to evaluate these findings in more ethnically and racially diverse populations are necessary.

## Conflict of interest statement

Dr. Scarmeas reports personal fees from Merck Consumer Health, Eisai, and personal fees from NIH unrelated to this manuscript. Dr. Seshadri reports consulting for Eisai and Biogen unrelated to this manuscript. On behalf of all authors, the corresponding author states that there is no conflict of interest.

## Appendix Supplementary materials

Image, application 1
