# Supplementary material for: Genetic predisposition to high circulating levels of interleukin 6 and risk for Alzheimer's disease. Discovery and replication
Source: J Prev Alzheimers Dis. 2025 Jan 1;12(1):100018. doi: 10.1016/j.tjpad.2024.100018 (PMC12184065; doi:10.1016/j.tjpad.2024.100018)

# **eMethods 1: Genotyping and imputation in HELIAD**

Genome-wide genotyping was performed at three different centers (the Centre National de Recherche en Génomique Humaine [CNRGH, Evry, France], the Life&brain center [Bonn, Germany], and the Erasmus Medical University [Rotterdam, The Netherlands]) using the Illumina Infinium Global Screening Array, as part of the European Alzheimer & Dementia Biobank (EADB) project. Base calling of raw reads was performed at CNRGH.

Variants included in the removal marker list by Illumina were excluded, and only variants for which the full-length probes aligned uniquely on GRCh38 genome without mismatches were kept. Variant intensity quality control (QC) was conducted for all autosomal variants according to established thresholds, and sex-check was performed using chromosome X variants.^1^ Subsequently, sample QC was performed using the PLINK v1.9 software. Samples with a missingness rate of more than 5%, sex inconsistencies, or with a heterozygosity rate deviating more than six standard deviations (SD) from the mean, were excluded. To identify population outliers, Principal Component Analysis (PCA) using the 1000 Genome Project phase 3 reference panel was performed, and the combined dataset was projected onto two dimensions, using the flashPCA2 software.^2^ To control for cryptic relatedness, one individual from each pair of samples with a kinship coefficient of more than 0.125 (cut-off for second-degree relatives) was excluded. Furthermore, variants with a missingness rate of more than 5% in at least one genotyping center, or with a significant differential missingness test (p <10^-10^), were excluded. The Hardy-Weinberg equilibrium test (p <5*10^-6^) was performed only in controls, and for each genotyping center/country separately.

To improve imputation accuracy, imputed variant frequencies were compared against two reference panels, the population of the Haplotype Reference Consortium v1.1 (HRC),^3^ excluding samples from the 1000 Genome Project, and the Genome Aggregation Database v3 (gnomAD),^4^ using the chi-square test. Variants with a x^2^ >3,000 in both HRC and gnomAD, or with a x^2^ >3,000 in one reference panel and not present in the other, were excluded. Finally, genome-wide association studies (GWAS) were performed between controls across genotyping centers to assess for potential frequency differences between genotyping centers, using the SNPTEST software,^5^ under an additive model and adjusting for associated principal components. Variants with a significant Likelihood Ratio Test at p <10^-5^ were excluded. Finally, ambiguous variants with a minor allele frequency (MAF) of ≤5% were removed, and only one copy of any duplicated variants was retained, prioritizing the one with the lowest missingness rate.

Samples and variants satisfying the aforementioned QC metrics were imputed on the Michigan Imputation Server (v1.2.4),^6^ using the TOPMed Freeze 5 reference panel. Phasing and imputation were performed using the EAGLE v2.4 and Minimac4 v4-1.0.2 software, respectively.

Apolipoprotein E isoforms were determined by the coding SNPs rs429358 and rs7412 genotypes, using the SNP array data.

# **eMethods 2: Adjusted survival curves**

We calculated adjusted survival curves separately for participants in different (low, intermediate, and high) IL-6 PRS categories using the conditional approach described by Therneau and colleagues.^7^ In this approach, subpopulations of interest are balanced in the following way. First, the data is replicated as many times as the levels of the stratifying variable (say k) by copying each row in the original data k times and assigning one copy to each stratum; this creates a new dataset which is balanced with respect to the stratifying variable since each stratum contains all the available combinations of the covariates of interest that are present in the data. Then, expected conditional (on the covariates of interest) survival curves are calculated for each stratum as the simple average of the individual curves for every hypothetical individual belonging in that stratum, based on the new artificial dataset.

# **eMethods 3: Replication sample**

## UK Biobank Participants

The UK Biobank (UKB, www.ukbiobank.ac.uk) is a very large, prospective, population-based cohort study that was established to investigate the determinants of health and disease, primarily in middle and old age. The study recruited more than half a million participants aged 40 to 69 years between 2006 and 2010 in the UK.^8^ At the baseline assessment, participants were evaluated at centers across England, Scotland, and Wales, and they provided electronically signed consent. The UK Biobank received ethical approval from the National Health Service (NHS) North West Centre for Research Ethics Committee (Ref: 11/NW/0382).

For the purposes of the present analysis, the following inclusion criteria were applied: (i) ≥ 60 years of age (to more closely match the age range of the HELIAD sample, and ensure the study population consisted of individuals at risk for developing late-onset dementia^9^), (ii) no prevalent Alzheimer’s disease (AD), (iii) satisfying the population structure control criteria (described below), (v) available hospital admissions or death registry data and International Classification of Diseases version 10 (ICD-10) diagnostic codes (to increase the accuracy of AD diagnosis). Exclusion criteria were: (i) prevalent AD, (ii) genetic relatedness (to increase the population-level generalizability of the findings^10^), and (iii) informed consent withdrawal at any point during the follow-up period.

## Diagnostic criteria and follow-up time calculation in the UKB

AD cases and controls were identified using hospital inpatient admission records and death registry data available from the Hospital Episode Statistics for England, the Scottish Morbidity Record for Scotland, and the Patient Episode Database for Wales. Primary and secondary hospital diagnoses were recorded using the ICD-10 coding system. Cases of AD were determined according to the following ICD-10 codes: G30 (AD), G30.0 (AD with early onset), G30.1 (AD with late onset), G30.8 (Other AD), G30.9 (AD, unspecified), F00 (Dementia in AD), F00.0 (Dementia in AD with early onset), F00.1 (Dementia in AD with late onset), F00.2 (Dementia in AD), and F00.9 (Dementia in AD, atypical or mixed type).

In the UKB, follow-up for disease outcomes is achieved via linkages to routinely-collected coded clinical healthcare datasets.^11^ UKB receives regularly updated linkages to national hospital admissions, cancer and mortality data for all participants. The follow-up time (in years) for each individual was calculated as the interval from the baseline assessment date until the date of first incident AD diagnosis, date of death, date of loss to follow-up, or end of follow-up, whichever occurred first. End of complete follow-up was based on the availability of electronic health record data in the UK Biobank, which was censored on 10/31/2022 for England, 08/31/2022 for Scotland, and 05/31/2022 for Wales.^9^

## Genotyping, imputation, and polygenic risk score calculation in the UKB

The protocols followed for genotyping and imputation in the UKB have been described elsewhere.^12,13^ To minimize the variability due to population structure in our dataset, we restricted our analyses to unrelated individuals based on the following previously proposed criteria^14,15^: (i) same reported and genetic sex, (ii) no putative sex aneuploidy, (iii) not marked as outliers for heterozygosity and missing rates, (iv) used to compute principal components, (v) at most 10 putative third-degree relatives, (vi) self-reported ‘White British’ race/ethnicity that was aligned with the PCA-assessed genetic ancestry.

The imputed genotype data v3 of UKB were used for interleukin 6 (IL-6) polygenic risk score (PRS) calculation, after excluding multi-allelic variants, those with imputation quality score < 0.4,^16^ and variants not present in HELIAD participants, according to the methodology described in section 2.3. of the main text. Specifically, the PRSice software was used to calculate IL-6 PRS scores for each participant at 10 a priori-defined genome-wide significance thresholds (i.e., 5*10^-8^, 0.0001, 0.001, 0.01, 0.05, 0.1, 0.2, 0.3, 0.4, and 0.5), by the clumping and thresholding (C + T) method.^17^ Higher PRS scores reflect greater genetic predisposition for elevated plasma levels of IL-6.

## Covariate assessment in the UKB

At the baseline assessment, information on sociodemographic, lifestyle, environmental, and health-related factors were collected through a touch-screen questionnaire and a nurse-led verbal interview, various physical examinations were performed, and biological samples were collected. We leveraged previous genomic studies that have imputed years of education by mapping each educational qualification category reported in the UKB dataset to an international standard classification of education category (ISCED), to categorize participants into three groups according to their completed years of education (< 10, between 10 and 15, and >15).^18,19^ Apolipoprotein E isoforms were determined by the coding SNPs rs429358 and rs7412 genotypes.

# **eResults 1: Missing data analysis in HELIAD**

There was a total of 1,699 participants without dementia or amnestic mild cognitive impairment (MCI) at baseline (eFigure 1). Of those, genotypic data were available for 1,017 unrelated participants. Participants with available genotypic data were slightly older, less educated, had a higher vascular burden score (VBS), and a higher proportion of them were men and from the Larissa center, compared to those without available genotypic data (n=682, eTable 2). Non-amnestic MCI prevalence was identical in those with and those without available genotypic data.

Participants with available cognitive follow-up (n=622) were younger and more educated compared to those without available follow-up (n=395, eTable 3). There were no differences in sex, study center, VBS, *APOE* ε4 allele carrier status, or the presence of non-amnestic MCI between participants with and those without available follow-up; most importantly, there were no differences in IL-6 PRS scores.

# **eReferences**

1. Grove ML, Yu B, Cochran BJ, et al. Best practices and joint calling of the HumanExome BeadChip: the CHARGE Consortium. *PLoS One.* 2013;8(7):e68095.

2. Abraham G, Qiu Y, Inouye M. FlashPCA2: principal component analysis of Biobank-scale genotype datasets. *Bioinformatics (Oxford, England).* 2017;33(17):2776-2778.

3. McCarthy S, Das S, Kretzschmar W, et al. A reference panel of 64,976 haplotypes for genotype imputation. *Nature genetics.* 2016;48(10):1279-1283.

4. Karczewski KJ, Francioli LC, Tiao G, et al. The mutational constraint spectrum quantified from variation in 141,456 humans. *Nature.* 2020;581(7809):434-443.

5. Marchini J, Howie B, Myers S, McVean G, Donnelly P. A new multipoint method for genome-wide association studies by imputation of genotypes. *Nature genetics.* 2007;39(7):906-913.

6. Das S, Forer L, Schönherr S, et al. Next-generation genotype imputation service and methods. *Nature genetics.* 2016;48(10):1284-1287.

7. Therneau T, Crowson C, Atkinson E. Adjusted Survival Curves'. 2015. <https://cran.r-project.org/web/packages/survival/vignettes/adjcurve.pdf>.

8. Sudlow C, Gallacher J, Allen N, et al. UK biobank: an open access resource for identifying the causes of a wide range of complex diseases of middle and old age. *PLoS medicine.* 2015;12(3):e1001779.

9. Qureshi D, Collister J, Allen NE, Kuźma E, Littlejohns T. Association between metabolic syndrome and risk of incident dementia in UK Biobank. *Alzheimer's & Dementia.* 2023;n/a(n/a).

10. Collister JA, Liu X, Clifton L. Calculating Polygenic Risk Scores (PRS) in UK Biobank: A Practical Guide for Epidemiologists. *Front Genet.* 2022;13:818574.

11. Wilkinson T, Schnier C, Bush K, et al. Identifying dementia outcomes in UK Biobank: a validation study of primary care, hospital admissions and mortality data. *European journal of epidemiology.* 2019;34(6):557-565.

12. Bycroft C, Freeman C, Petkova D, et al. The UK Biobank resource with deep phenotyping and genomic data. *Nature.* 2018;562(7726):203-209.

13. Collins R. What makes UK Biobank special? *Lancet.* 2012;379(9822):1173-1174.

14. Sinnott-Armstrong N, Tanigawa Y, Amar D, et al. Genetics of 35 blood and urine biomarkers in the UK Biobank. *Nat Genet.* 2021;53(2):185-194.

15. Tanigawa Y, Li J, Justesen JM, et al. Components of genetic associations across 2,138 phenotypes in the UK Biobank highlight adipocyte biology. *Nature Communications.* 2019;10(1):4064.

16. Zheng HF, Ladouceur M, Greenwood CM, Richards JB. Effect of genome-wide genotyping and reference panels on rare variants imputation. *Journal of genetics and genomics = Yi chuan xue bao.* 2012;39(10):545-550.

17. Choi SW, O'Reilly PF. PRSice-2: Polygenic Risk Score software for biobank-scale data. *GigaScience.* 2019;8(7).

18. Lee JJ, Wedow R, Okbay A, et al. Gene discovery and polygenic prediction from a genome-wide association study of educational attainment in 1.1 million individuals. *Nat Genet.* 2018;50(8):1112-1121.

19. Amin V, Fletcher JM, Sun Z, Lu Q. Higher educational attainment is associated with longer telomeres in midlife: Evidence from sibling comparisons in the UK Biobank. *SSM - Population Health.* 2022;17:101018.

| **eTable 1:** Number of SNPs included in IL-6 PRS calculation at each of the 10 a-priori-defined P value thresholds for genome-wide significance. | |
| --- | --- |
| GWAS P value threshold | Number of SNPs |
| 5e-8 | 5 |
| 0.0001 | 128 |
| 0.001 | 906 |
| 0.01 | 6723 |
| 0.05 | 24702 |
| 0.1 | 41632 |
| 0.2 | 67438 |
| 0.3 | 87429 |
| 0.4 | 103213 |
| 0.5 | 116338 |
| Abbreviations: SNP, single nucleotide polymorphism; IL-6, interleukin 6; PRS, polygenic risk score; GWAS, genome-wide association study. | |

| **eTable 2:** HELIAD participant baseline characteristics by genotypic data availability. | | | | |
| --- | --- | --- | --- | --- |
|  | Total  (1,699) | Available  genotypic data  (1,017) | Not available  genotypic data  (682) | P value |
| Age, years, mean (SD) | 73.4 (5.2) | 73.9 (5.2) | 72.8 (5.2) | **<0.001 ^a^** |
| Sex, n (%)  Women  Men | 1017 (60)  682 (40) | 585 (58)  432 (42) | 432 (63)  250 (37) | **0.02 ^a^** |
| Study center, n (%)  Larissa  Marousi | 1251 (74)  448 (26) | 920 (90)  97 (10) | 331 (49)  351 (51) | **<0.001 ^a^** |
| Education, years, median (IQR) | 6 (5, 12) | 6 (5, 9) | 11 (6, 14) | **<0.001 ^a^** |
| Vascular burden score, median (IQR) | 2 (1, 2) | 2 (1, 2) | 1.5 (1, 2) | **0.007 ^a^** |
| Non-amnestic MCI, n (%)  No  Yes | 1607 (95)  92 (5) | 962 (95)  55 (5) | 645 (95)  37 (5) | 0.99 |
| Abbreviations: HELIAD, Hellenic Longitudinal Investigation of Aging and Diet; SD, standard deviation; IQR, interquartile range; MCI, mild cognitive impairment.  ^a^ Significant at p ≤.05. | | | | |

| **eTable 3:** HELIAD participant baseline characteristics by cognitive follow-up data availability. | | | | |
| --- | --- | --- | --- | --- |
|  | Total  (1017) | Available  follow-up data  (622) | Not available  follow-up data  (395) | P value |
| Age, years, mean (SD) | 73.9 (5.2) | 73.4 (5.0) | 74.7 (5.3) | **<0.001 ^a^** |
| Sex, n (%)  Women  Men | 585 (58)  432 (42) | 363 (58)  259 (42) | 222 (56)  173 (44) | 0.50 |
| Study center, n (%)  Larissa  Marousi | 920 (90)  97 (10) | 556 (89)  66 (11) | 364 (92)  31 (8) | 0.14 |
| Education, years, median (IQR) | 6 (5, 9) | 6 (5, 11) | 6 (4, 7) | **<0.001 ^a^** |
| Vascular burden score, median (IQR) | 2 (1, 2) | 2 (1, 2) | 2 (1, 2) | 0.29 |
| *APOE* ε4 allele carrier, n (%)  No  Yes | 859 (84)  158 (16) | 524 (84)  98 (16) | 335 (85)  60 (15) | 0.81 |
| Non-amnestic MCI  No  Yes | 962 (95)  55 (5) | 593 (95)  29 (5) | 369 (93)  26 (7) | 0.19 |
| PRS for IL-6 * 10^-4^, mean (SD)  GWAS threshold 5*10^-8^  GWAS threshold 0.0001  GWAS threshold 0.001  GWAS threshold 0.01  GWAS threshold 0.05  GWAS threshold 0.1  GWAS threshold 0.2  GWAS threshold 0.3  GWAS threshold 0.4  GWAS threshold 0.5 | -434.27 (204.08)  -1.24 (20.18)  9.71 (6.41)  -3.21 (2.04)  0.36 (0.94)  -0.60 (0.68)  0.57 (0.49)  -0.07 (0.40)  0.12 (0.35)  0.10 (0.31) | -437.91 (204.13)  -1.47 (20.08)  9.74 (6.52)  -3.25 (2.00)  0.33 (0.91)  -0.62 (0.65)  0.55 (0.47)  -0.09 (0.38)  0.10 (0.34)  0.09 (0.31) | -428.54 (204.12)  -0.87 (20.34)  9.67 (6.25)  -3.15 (2.11)  0.41 (0.97)  -0.57 (0.71)  0.60 (0.51)  -0.05 (0.42)  0.14 (0.36)  0.12 (0.32) | 0.48  0.64  0.87  0.48  0.15  0.18  0.18  0.14  0.11  0.12 |
| Abbreviations: HELIAD, Hellenic Longitudinal Investigation of Aging and Diet; SD, standard deviation; IQR, interquartile range; MCI, mild cognitive impairment PRS, polygenic risk score; IL-6, interleukin 6; GWAS, genome-wide association study.  ^a^ Significant at p ≤.05. | | | | |

| **eTable 4:** UK Biobank participant baseline characteristics by AD incidence. | | | | |
| --- | --- | --- | --- | --- |
|  | Total  (142,637) | No AD at follow-up  (139,900) | AD at follow-up  (2,737) | P value |
| Age, years, mean (SD) | 64.2 (2.8) | 64.2 (2.8) | 65.7 (2.7) | **< 0.001** |
| Sex, n (%)  Women  Men | 73,707 (52)  68,930 (48) | 72,302 (52)  67,598 (48) | 1,405 (51)  1,332 (49) | 0.72 |
| Education, n (%)  Less than 10 years  Between 10 to 15 years  More than 15 years | 40,524 (28)  48,780 (34)  53,333 (37) | 39,462 (28)  47,937 (34)  52,501 (38) | 1,062 (39) ^b^  843 (31) ^b^  832 (30) ^b^ | **< 0.001** |
| *APOE* ε4 allele carrier, n (%)  No  Yes | 101,832 (71)  40,805 (29) | 100,831 (72)  39,069 (28) | 1,001 (37)  1,736 (63) | **< 0.001** |
| PRS for IL-6 * 10^-4^, mean (SD)  GWAS threshold 5*10^-8^  GWAS threshold 0.0001  GWAS threshold 0.001  GWAS threshold 0.01  GWAS threshold 0.05  GWAS threshold 0.1  GWAS threshold 0.2  GWAS threshold 0.3  GWAS threshold 0.4  GWAS threshold 0.5 | -387.98 (203.99)  10.25 (23.44)  18.07 (7.12)  -2.83 (2.20)  0.64 (1.01)  -0.53 (0.72)  0.72 (0.51)  0.11 (0.42)  0.22 (0.36)  0.25 (0.33) | -387.96 (203.96)  10.25 (23.44)  18.07 (7.13)  -2.83 (2.21)  0.64 (1.01)  -0.53 (0.72)  0.72 (0.51)  0.11 (0.42)  0.22 (0.36)  0.25 (0.33) | -388.94 (205.81)  10.04 (23.78)  18.08 (6.97)  -2.74 (2.13)  0.70 (0.97)  -0.48 (0.71)  0.76 (0.51)  0.14 (0.41)  0.25 (0.36)  0.27 (0.32) | 0.8  0.64  0.94  **0.03**  **<0.001**  **<0.001**  **<0.001**  **<0.001**  **<0.001**  **<0.001** |
| Statistically significant findings at p ≤ 0.05 are indicated in bold.  Abbreviations: AD, Alzheimer’s disease; SD, standard deviation; PRS, polygenic risk score; IL-6, interleukin 6; GWAS, genome-wide association study.  ^b^ Significantly different column proportion compared to participants with no AD at follow-up. | | | | |

| **eTable 5:** Associations between PRS for IL-6 and AD incidence accounting for death as a competing event in the UK Biobank. | | | | | |  |
| --- | --- | --- | --- | --- | --- | --- |
| PRS for IL-6 | | At risk, n | SHR (95% CI) ^a^ | Raw p value | Adjusted p value |  |
| Genome-wide significance threshold | 5*10^-8^ | 142,637 | 0.99 (0.96 - 1.03) | 0.724 | 0.805 |  |
|  | 0.0001 |  | 0.99 (0.95 - 1.03) | 0.586 | 0.732 |  |
|  | 0.001 |  | 1.00 (0.97 - 1.04) | 0.916 | 0.916 |  |
|  | 0.01 |  | 1.04 (1.00 - 1.08) | 0.037 | 0.052 |  |
|  | 0.05 | Events, n | **1.06 (1.02 - 1.10)** | **0.001** | **0.003 ^c^** |  |
|  | 0.1 |  | **1.06 (1.02 - 1.10)** | **0.001** | **0.003 ^c^** |  |
|  | 0.2 | 2,737 | **1.08 (1.04 - 1.12)** | **<0.001** | **0.001 ^c^** |  |
|  | 0.3 |  | **1.07 (1.03 - 1.11)** | **<0.001** | **0.002 ^c^** |  |
|  | 0.4 |  | **1.06 (1.02 - 1.10)** | **0.002** | **0.003 ^c^** |  |
|  | 0.5 |  | **1.06 (1.02 - 1.10)** | **0.002** | **0.004 ^c^** |  |
| Results from Fine-Gray subdistribution hazard models with AD as the primary outcome of interest and death as a competing event.  ^a^ Values represent subdistribution hazard ratios of IL-6 PRS for AD, accounting for death as a competing event.  ^b^ Models were adjusted for baseline age, sex, education, *APOE* ε4 carrier status, and the first two principal components for population structure.  ^c^ Significant after false-discovery rate control at <5% using the Benjamini-Hochberg procedure.  Abbreviations: PRS, polygenic risk score; IL-6, interleukin 6; AD, Alzheimer’s disease; SHR, subdistribution hazard ratio; CI, confidence interval. | | | | | |  |

**eFigure 1:** Some of the potential biological pathways linking interleukin 6 (IL-6) to Alzheimer’s disease (AD) pathogenesis.


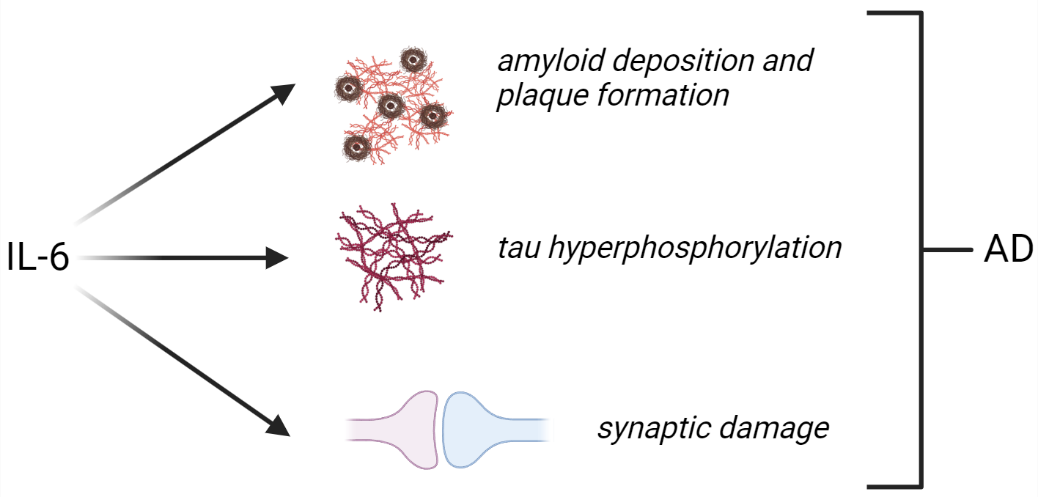

Supplement: Supplementary file 1 [file mmc1.docx]
